# Anti-Phospholipase A2 Receptor Antibody Expression at Different Stages of Idiopathic Membranous Nephropathy

**DOI:** 10.1155/2022/5962195

**Published:** 2022-07-30

**Authors:** Keying Fu, Pei Zhang, Yeguang Han, Ru Wang, Junhong Cai

**Affiliations:** Medical Laboratory Center of Hainan General Hospital, Hainan Affiliated Hospital of Hainan Medical University, Haikou 570311, China

## Abstract

The significance of blood anti-phospholipase A2 receptor (PLA_2_R) antibodies in the diagnosis of different stages of idiopathic membranous nephropathy (IMN) was investigated. The expression and distribution of anti-PLA_2_R antibodies in renal biopsy tissue of patients with different stages of IMN were examined by immunohistochemistry. In addition, blood anti-PLA_2_R antibodies were determined by indirect immunofluorescence for the same patients, and the results were compared with the anti-PLA_2_R antibody expression in renal biopsy tissue. The positive fluorescence intensities of IMN stages I, IV, and V were mostly ± or + (40/80). There was no significant difference in fluorescence titer between these stages (*p* > 0.05). These results were consistent with the immunohistochemistry results, and the kappa statistic was 0.95. The positive fluorescence intensities of IMN stages II and III were mostly ++ to ++++ (33/60). There was no significant difference in fluorescence intensities between these two stages (*p* > 0.05), but there was a significant difference in fluorescence intensities between stages II and III and stages I, IV, and V (*p* < 0.001). These results were consistent with the immunohistochemistry results, and the kappa statistic was 0.97 (*p* < 0.001). Therefore, blood anti-PLA_2_R levels were positively correlated with anti-PLA_2_R expression in renal biopsy tissue in patients with different stages of IMN. In addition, the fluorescence intensities of IMN stages II and III were significantly different from those of stages I, IV, and V. Therefore, blood anti-PLA_2_R levels can be used for in vitro differential diagnosis and the monitoring of treatment, as it can distinguish stage II; and III; from stage I, IV, and V IMN.

## 1. Introduction

Idiopathic membranous nephropathy (IMN) is a common primary glomerular disease characterized by massive proteinuria or nephrotic syndrome [1]. It is associated with long-term risk of renal failure, as well as thrombosis and cardiovascular complications. Typical pathological features are glomerular subepithelial deposition and intramembranous immune complex deposition, with segmental or global sclerosis. Immunopathological examination shows fine granular deposition of IgG and C3 along the basement membrane. Electron microscopy shows electron-dense deposits in different parts of the basement membrane. The basement membrane lesions are classified into five stages of severity.

- Stage I: the glomerular lesions are mild. PASM staining shows ribbon-shaped vacuolar degeneration of the basement membrane, similar to minimal change glomerulopathy, and sometimes Masson staining shows a small amount of subepithelial eosinophilic deposits.
- Stage II: diffuse thickening of the basement membrane. Masson staining shows a large amount of subepithelial eosinophilic deposits, and PASM staining shows spikes projecting from the basement membrane.
- Stage III: severe diffuse thickening of the basement membrane, stenosis of the capillary lumen, and mild to moderate diffuse proliferation of mesangial cells and stroma; segmental or global sclerosis is seen in severe cases. Masson staining shows a large number of eosinophilic deposits in the basement membrane, and PASM staining shows double-track or ring-like changes of the basement membrane.
- Stage IV: there are two types of changes. (i) Immune complex deposition stops, and deposited immune complexes are gradually absorbed; most cases are advanced from stage I or II; only irregular thickening of the basement membrane is seen. Masson staining shows a small number of eosinophilic deposits, and PASM staining shows segmental thickening of the basement membrane. (ii) A deterioration from stage III, manifested as global and segmental sclerosis.
- Stage V: the glomeruli return to normal [2].

Before the identification of the autoantigen phospholipase A2 receptor (PLA_2_R) in 2009, IMN was mainly diagnosed by renal biopsy, histological examination, or electron microscopy [3]. However, histopathological examination alone cannot fully distinguish IMN from membranous nephropathy (MN) from other causes. In addition, it is often difficult to distinguish between the different stages due to objective factors such as material selection. Moreover, as histopathological examination is an invasive procedure, many patients do not receive timely diagnosis due to contraindications to biopsy, psychological disorders, or other reasons and consequently do not receive timely standard treatment. In the present study, we explored the significance of the correlation between anti-PLA_2_R in blood and anti-PLA_2_R expression in renal biopsy tissue in the diagnosis of different stages of IMN.

## 2. Materials and Methods

### 2.1. Subjects

A total of 120 renal biopsy specimens (embedded in paraffin at room temperature) were collected from patients in Hainan General Hospital. Patients' ages ranged from 14 to 76 years (average 42 years); 100 patients had IMN (*n* = 20 for each stage), 10 had lupus nephritis, and 10 had hepatitis B-related nephritis. Blood samples were collected from the same patients as well as 20 healthy blood donors (samples were stored at –80°C). The renal biopsy tissue was stained with HE, PAS, PASM, and Masson trichrome and examined for pathological changes using immunofluorescence and electron microscopy. Inclusion criteria were as follows: patients diagnosed with IMN based on renal pathology using light microscopy, electron microscopy, and immunofluorescence, combined with clinical assessment, were included. Exclusion criteria were as follows: lupus nephritis; hepatitis B-related nephritis; tumor-related MN; and other diseases that may cause MN.

### 2.2. Groups

Patients with IMN according to pathological diagnosis by light and electron microscopy were defined as the experimental group. Patients with secondary MN (lupus nephritis and hepatitis B-related nephritis) were defined as the control group. The blood samples were divided into three groups: the experimental group, the control group, and the normal group.

### 2.3. Methods

The expression and distribution of anti-PLA_2_R in the renal biopsy tissue of each group were analyzed by immunohistochemical staining of the tissue using the streptavidin-biotin peroxidase complex (SABC) technique. The SABC reagent was purchased from Beijing Zhongshan Jinqiao Biological Technology Co. Ltd. (China), and anti-PLA_2_R was acquired from Abcam (USA).

Blood anti-PLA_2_R levels were measured by indirect immunofluorescence, and the values were compared with anti-PLA_2_R expression in the renal biopsy tissue for each group and at each stage. Indirect immunofluorescence was performed using EU90 cells coated with non-transfected and transfected PLA_2_R. The kit was purchased from EUROIMMUN Medical Laboratory Diagnostics Stock Company (Germany) and was used as a semi-quantitative method according to the instructions given: titer 1 : 10 equals ±, titer 1 : 32 equals +, titer 1 : 100 equals ++, titer 1 : 320 equals ++, and titer 1 : 1000 equals ++++.

The sensitivity and specificity of the results for each group were calculated. Chi-square and kappa agreement tests were performed using SPSS 18.0. The positive, negative, and total accuracy were calculated.

## 3. Results

The expression of anti-PLA_2_R in the renal biopsy tissue and the anti-PLA_2_R fluorescence intensity in blood are shown in Table 1.

The positive fluorescence intensities of stages I, IV, and V were mostly ± to + (40/80). There was no significant difference in fluorescence titer between these stages (*p* > 0.05). These results were consistent with immunohistochemistry, and the kappa statistic was 0.95 (*p* < 0.001) (Table 2).

The positive fluorescence intensities of stages II and III were mostly ++ to ++++ (33/60). There was no significant difference in fluorescence titer between the two stages (*p* > 0.05), but there was a significant difference with those of stages I, IV, and V (*p* < 0.001) (Table 3). These results were consistent with the immunohistochemistry, and the kappa statistic was 0.97 (Table 4).

## 4. Discussion

IMN is an autoimmune disease affecting specific organs. It is a common cause of nephrotic syndrome in adults and can occur at any age. It was previously thought that all cases of immune complex nephritis were due to passive deposition of circulating immune complexes in the glomerulus. However, research in recent years has changed this understanding. It has been demonstrated in an animal model of IMN that circulating antibodies binding to podocyte antigens led to in situ immune complex deposition on the epithelial side [4].

PLA_2_R is a type I transmembrane receptor and one of the four members of the mammalian mannose receptor family. The N-terminus of PLA_2_R is a cysteine-rich domain. Adjacent to the N-terminus is a fibronectin type II domain and eight tandem C-type lectin-like domains (CTLD). The C-terminus in the cytoplasm contains structures that interact with the endocytic apparatus, which may be involved in the transport of extracellular ligand molecules into the cell. PLA_2_R is mainly divided into two types: M-type and N-type. It has been shown that M-type PLA_2_R is the main target antigen of autoantibodies [5].

In IMN patients, anti-PLA_2_R in blood binds to a conformation-dependent epitope of PLA_2_R to form in situ immune complexes deposited on the glomerular basement membrane, causing damage to podocytes, thickening of the glomerular basement membrane, and changes in permeability [6]. PLA_2_R is highly expressed in glomerular podocytes and co-localizes with IgG4 in the glomerular immune complex deposits of IMN patients. Anti-PLA_2_R antibodies in IMN patients are predominantly of the IgG4 subtype, which is the predominant immunoglobulin subclass in glomerular deposits. IgG eluted from deposits of IMN patients recognizes PLA_2_R. Thus, PLA_2_R is a major antigen leading to IMN. In 2009, Beck et al. [7] detected M-type PLA_2_R in 26 of 37 patients with IMN using Western blotting, suggesting that detection of anti-PLA_2_R in circulating blood is likely to play an important role in diagnosing and monitoring IMN activity. IgG4 is the most important subtype of anti-PLA_2_R in IMN, which is different from secondary MN (e.g. lupus MN or malignancy-associated MN), where the main subtypes are IgG2 and IgG3. It has been shown that the anti-PLA_2_R titer is related to IMN activity and can also be used as an indicator of therapeutic efficacy [8].

It has been reported that immunohistochemistry can be used to detect anti-PLA_2_R [9]. Qin et al. [10] suggested that anti-PLA_2_R has high specificity for IMN, and its antibody titer in blood can be used to measure the therapeutic efficacy and prognosis of patients. Zhou et al. [11] suggested that anti-PLA_2_R may be specific pathogenic antibodies of IMN. Guan et al. [12] found a high serum anti-PLA_2_R antibody positive rate in IMN patients and suggested that combined detection of serum anti-PLA_2_R antibodies and glomerular IgG4 subtype is helpful for the diagnosis and differential diagnosis of MN etiology. Indirect immunofluorescence using transfected cells has been reported to be a convenient and reliable method for hematological screening for anti-PLA_2_R [9]. It is suitable for the qualitative and quantitative analysis of anti-PLA_2_R [13–17].

Hofstra et al. [18] suggested that anti-PLA_2_R levels can be used to monitor IMN activity and treatment outcomes. Anti-PLA_2_R is a useful biomarker for the diagnosis of primary MN and can be used to monitor disease activity and predict remission or relapse. Anti-PLA_2_R positivity was observed in 50–80% of MN patients, depending on disease activity. The sensitivity of anti-PLA_2_R detection depends on the biological and population differences of the disease rather than the specificity of the analytical method. Hence, anti-PLA_2_R positivity is diagnostic for patients with contraindications to renal biopsy, such as those on anticoagulation to treat thrombosis, or those with only one kidney (solitary kidney).

In the early stages of disease, when anti-PLA_2_R antibodies are produced and bind to antigens on podocytes, immune complex deposits containing anti-PLA_2_R antigen-antibody complexes are formed, which induces podocyte damage and proteinuria. At this time, serum anti-PLA_2_R cannot be detected by stable methods because the kidneys act as a sink to absorb all circulating antigen-antibody complexes. In these cases, definitive diagnosis can only be made by the detection of PLA_2_R deposits in immunofluorescent stained renal biopsy tissue. With the development of immune activity and disease, renal absorption of anti-PLA_2_R antibodies gradually ceases, and serum anti-PLA_2_R can be detected and used as a tool to diagnose and monitor disease activity. When a patient enters remission, anti-PLA_2_R disappears from the serum, although proteinuria is not yet resolved. At this time, immune complex deposits are still detected in renal biopsy tissue [19].

Numerous studies conducted over the past 3 years have shown that anti-PLA_2_R levels correlate with urinary protein excretion and disease activity. Antibody levels are undetectable during spontaneous remission or treatment-induced remission and reappear or increase during relapses. Antibody levels also predict prognosis, as high antibody titers are associated with a reduced risk of spontaneous remission or immunosuppression-induced remission and an increased risk of nephrotic syndrome and worsening renal function in non-nephrotic patients. The interval between initiation of immunosuppressive therapy and remission was significantly increased in patients in the group with the highest antibody titers. In response to this issue, a research team led by Zhihong Liu at the General Hospital of Eastern Theater Command conducted a study in 572 patients with IMN confirmed by renal biopsy [20]. The results suggested that glomerular PLA_2_R antigen is more reliable than serum anti-PLA_2_R for the diagnosis of IMN, whereas serum anti-PLA_2_R may be more reliable than glomerular PLA_2_R antigen for measuring disease activity and renal function. Therefore, combined detection of serum anti-PLA_2_R and glomerular PLA_2_R antigen deposits may provide more empirical evidence for diagnosis, treatment response, and relapses in patients with IMN. In the study, the positive fluorescence intensities of stages I, IV, and V were mostly ± to+ (40/80), with no significant difference in fluorescence titer between these stages. These results were consistent with the immunohistochemistry, and the kappa statistic was 0.95. The positive fluorescence intensities of stages II and III were mostly ++ to ++++. There was no significant difference in fluorescence intensity between these two stages, but there was a significant difference with the fluorescence intensity of stages I, IV, and V (*p* < 0.001). These results were consistent with the immunohistochemistry, and the kappa statistic was 0.97. These findings also support the above conclusion. Hence, blood anti-PLA_2_R measurements are useful for in vitro differential diagnosis and the monitoring of treatment, as it can distinguish stage II and III from stage I, IV, and V IMN. Anti-PLA_2_R can reflect the progress of IMN, judge the treatment effect and prognosis of patients to a certain extent, and provide help for the individualized treatment strategy of IMN patients with different stages. Also, it will pay more attention to the stage II and III IMN patients with high titer of anti-PLA_2_R.

In addition, the present study showed that MN occurs mostly (65%) at ages 30–60 years, and that the proportion of MN in kidney disease is on the rise, which is consistent with the findings of other studies [21,22]. Therefore, patients in this age group should receive sufficient attention.

## 5. Conclusion

Blood anti-PLA_2_R detected by indirect immunofluorescence using EU90 cells coated with non-transfected and transfected PLA_2_R can be used as a specific indicator for in vitro diagnosis of IMN. The method facilitates the differential diagnosis of IMN and secondary MN and, more importantly, the differential diagnosis and treatment monitoring of different stages of IMN.

## Figures and Tables

**Table 1:** Anti-PLA_2_R expression in tissue and anti-PLA_2_R fluorescence intensity in blood.

Group	Anti-PLA2R expression in tissue					Anti-PLA2R fluorescence intensity in blood				
	−	±	+	++	+++	−	±	+	++	+++
Stage I (20)	6	6	8	0	0	5	6	9	0	0
Stage II (20)	2	1	1	8	8	1	0	2	9	8
Stage III (20)	2	0	1	8	9	2	0	1	9	8
Stage IV (20)	6	6	8	0	0	5	6	9	0	0
Stage V (20)	8	8	4	0	0	8	9	3	0	0
Control group (20)	19	0	1	0	0	19	0	1	0	0
Normal group (20)	—	—	—	—	—	20	0	0	0	0
Total	43	21	21	21	21	60	21	25	25	16

**Table 2:** Correlation of blood anti-PLA_2_R levels with renal biopsy levels of stages I, IV, and V.

Blood	Renal biopsy		
	Positive (± to +)	Negative	Total
Positive (± to +)	41	0	41
Negative	2	37	39
Total	43	37	80

*x* ^2^=0.50, *p* > 0.05; *κ* = 0.95, *p* < 0.001.

**Table 3:** Comparison of blood anti-PLA_2_R fluorescence intensity between stages II and III and stages I, IV, and V.

Group	± to +	++ to +++	Total
Stages I, IV, and V	42	0	42
Stages II and III	3	34	37
Total	45	34	79

*x* ^2^=68.76, *p* < 0.001.

**Table 4:** Correlation of blood anti-PLA_2_R levels with renal biopsy levels of stages II and III.

Blood	Renal biopsy		
	Positive (++ to +++)	Negative	Total
Positive (++ to +++)	33	0	33
Negative	1	26	27
Total	34	26	60

*x* ^2^=0.00, *p* > 0.05; *κ* = 0.97, *p* < 0.001.

## Data Availability

The underlying data supporting the results of our study can be found in Hospital Information System of Hainan General Hospital.
